# Directional Atherectomy Followed by a Paclitaxel-Coated Balloon to Inhibit Restenosis and Maintain Vessel Patency: Twelve-Month Results of the DEFINITIVE AR Study

**DOI:** 10.1161/CIRCINTERVENTIONS.116.004848

**Published:** 2017-09-15

**Authors:** Thomas Zeller, Ralf Langhoff, Krishna J. Rocha-Singh, Michael R. Jaff, Erwin Blessing, Beatrice Amann-Vesti, Marek Krzanowski, Patrick Peeters, Dierk Scheinert, Giovanni Torsello, Sebastian Sixt, Gunnar Tepe

**Affiliations:** From the Universitäts-Herzzentrum Bad Krozingen, Germany (T.Z.); Sankt Getrauden-Krankenhaus, Berlin, Germany (R.L.); Prairie Heart Institute at St. John’s Hospital, Springfield, IL (K.J.R.-S.); VasCore–the Vascular Ultrasound Core Laboratory, Massachusetts General Hospital, Boston (M.R.J.); SRH Klinikum Karlsbad-Langensteinbach, Germany (E.B.); Clinic for Angiology, University Hospital Zurich, Switzerland (B.A.-V.); Zakład Leczniczy Angio-Medicus, Krakow, Poland (M.K.); Department of Cardiovascular and Thoracic Surgery, Imelda Hospital, Bonheiden, Belgium (P.P.); Department of Interventional Angiology, University Hospital Leipzig, Germany (D.S.); University Hospital Muenster, Klinik for Vascular and Endovascular Surgery, Germany (G. Torsello); Swiss Cardiovascular Center, Division of Angiology, University Hospital, Inselspital Bern, Switzerland (S.S.); and Klinikum Rosenheim, Germany (G. Tepe).

**Keywords:** atherectomy, directional atherectomy, drug-eluting balloon, paclitaxel, peripheral artery disease, plaque modification

## Abstract

**Background—:**

Studies assessing drug-coated balloons (DCB) for the treatment of femoropopliteal artery disease are encouraging. However, challenging lesions, such as severely calcified, remain difficult to treat with DCB alone. Vessel preparation with directional atherectomy (DA) potentially improves outcomes of DCB.

**Methods and Results—:**

DEFINITIVE AR study (Directional Atherectomy Followed by a Paclitaxel-Coated Balloon to Inhibit Restenosis and Maintain Vessel Patency—A Pilot Study of Anti-Restenosis Treatment) was a multicenter randomized trial designed to estimate the effect of DA before DCB to facilitate the development of future end point-driven randomized studies. One hundred two patients with claudication or rest pain were randomly assigned 1:1 to DA+DCB (n=48) or DCB alone (n=54), and 19 additional patients with severely calcified lesions were treated with DA+DCB. Mean lesion length was 11.2±4.0 cm for DA+DCB and 9.7±4.1 cm for DCB (*P*=0.05). Predilation rate was 16.7% for DA+DCB versus 74.1% for DCB; postdilation rate was 6.3% for DA+DCB versus 33.3% for DCB. Technical success was superior for DA+DCB (89.6% versus 64.2%; *P*=0.004). Overall bail-out stenting rate was 3.7%, and rate of flow-limiting dissections was 19% for DCB and 2% for DA+DCB (*P*=0.01). One-year primary outcome of angiographic percent diameter stenosis was 33.6±17.7% for DA+DCB versus 36.4±17.6% for DCB (*P*=0.48), and clinically driven target lesion revascularization was 7.3% for DA+DCB and 8.0% for DCB (*P*=0.90). Duplex ultrasound patency was 84.6% for DA+DCB, 81.3% for DCB (*P*=0.78), and 68.8% for calcified lesions. Freedom from major adverse events at 1 year was 89.3% for DA+DCB and 90.0% for DCB (*P*=0.86).

**Conclusions—:**

DA+DCB treatment was effective and safe, but the study was not powered to show significant differences between the 2 methods of revascularization in 1-year follow-up. An adequately powered randomized trial is warranted.

**Clinical Trial Registration—:**

http://www.clinicaltrials.gov. Unique Identifier: NCT01366482.

**WHAT IS KNOWN**

- Drug-coated balloons (DCB) angioplasty of femoropopliteal TASC II A and B lesions (Trans-Atlantic Inter-Society Consensus Document of Management of Peripheral Arterial Disease) has shown promising mid-term results in randomized controlled trials. However, depending on lesion complexity, bail-out stent placement is indicated in a significant percentage of interventions, and patency failures occur in particular in calcified lesions.
- Vessel preparation with debulking devices, such as directional atherectomy (DA) might improve acute and longer-term technical outcomes of DCB angioplasty as suggested in small single-center studies.

**WHAT THE STUDY ADDS**

- Vessel preparation before DCB angioplasty using DA for the treatment of femoropopliteal artery disease is safe.
- Vessel preparation before DCB angioplasty using DA significantly improves acute treatment outcomes, such as residual stenosis, interventional success, and dissection rates.
- Despite significantly better acute outcomes, this underpowered prospective trial did not show superior mid-term technical and clinical outcomes for vessel preparation before DCB angioplasty using DA as compared with plain DCB angioplasty.
- Lesion calcification and lesion length >10 cm were identified as potential predictors for superior outcomes for the combination therapy. A larger-scale randomized controlled study focusing on such complex lesion types is warranted.

Peripheral artery disease (PAD) is a common disorder that affects >200 million people worldwide.^1^ PAD is a progressive disease and is associated with a significant reduction in patient quality of life. Approximately 10% to 15% of patients with claudication progress to critical limb ischemia for ≥5 years, putting them at risk for amputation.^2^

Given the lack of data comparing endovascular to open procedures, the TASC II (Trans-Atlantic Inter-Society Consensus Document of Management of Peripheral Arterial Disease) update released in 2015 did not provide recommendations on procedure type and nor did it recommend specific devices when treating PAD patients, also because of the paucity of data.^3^ The first generation methods of endovascular treatment of PAD include percutaneous transluminal angioplasty (PTA) and provisional bare metal stent placement. However, use of standalone PTA may result in plaque fracture, arterial wall stretching, and dissection.^4^ This can initiate the cycle of injury, healing, and negative remodeling, placing the patient at increased risk for restenosis.^5^ Stent placement can also result in unintended negative consequences because leaving a metal implant behind can decrease future treatment options or result in-stent fractures.^6^

To address some of these challenges, several published clinical studies have investigated the effectiveness of local administration of paclitaxel on restenosis after PTA of lesions located in the femoropopliteal artery segments.^7–10^ Results with paclitaxel drug-coated balloons (DCB) are promising in TASC II A and B lesions,^7–10^ and patients with long lesions had high 1-year patency in the SFA-LONG study (Drug Eluting Balloon [DEB] and Long Lesions of Superficial Femoral Artery [SFA] Ischemic Vascular Disease).^11^ However, calcified and longer lesions remain a challenging subset that is less responsive to DCBs, resulting in higher provisional stent rates.^12–14^

Another second generation device to treat PAD, directional atherectomy (DA), removes plaque from the vessel wall, providing improved luminal gain and plaque modification resulting in low rates of bail-out stenting, perforation, and dissection.^15^

As DA reduces plaque volume, this could potentially facilitate a more homogenous delivery of drug to the vessel wall and increase drug penetration.^16,17^

Both technologies were used together in relatively small, single-center studies to investigate whether DA and DCB could be an effective treatment for lower extremity PAD, and in particular, those lesions that are difficult to treat with one modality alone.^16,18–20^ This study aims to expand on these prior studies and generate hypotheses for future trials that combines treatment with both DA and DCB.

## Methods

### Study Design

DEFINITIVE AR study (Directional Atherectomy Followed by a Paclitaxel-Coated Balloon to Inhibit Restenosis and Maintain Vessel Patency—A Pilot Study of Anti-Restenosis Treatment) is a prospective, multicenter, randomized controlled pilot trial designed to assess and estimate the safety and the effect of treating vessels with DA before DCB angioplasty (DA+DCB) as compared with DCB angioplasty alone to facilitate the development of future randomized studies. This study evaluated lesions 7 to 15 cm long in femoropopliteal arteries in patients with claudication or rest pain (Rutherford clinical category [RCC], 2–4). The rationale for excluding shorter lesions was the excellent outcome of DCB angioplasty in these simple lesions in former DCB trials and for the upper length limit was to stay with 2 DCBs maximum guaranteeing full lesion coverage, including DCB overlap. Study assessments occurred at baseline, procedure, predischarge, 30 days, 6 months, and 1 year after the study procedure. The study was conducted in accordance with good clinical practices, ISO 14155:2011, and applicable laws of all relevant governing bodies. Each site’s ethics committee reviewed and approved the study protocol. All patients signed an informed consent form before any study activities took place. Regular monitoring visits were conducted at all investigational sites. A clinical events committee composed of independent physicians adjudicated all adverse events. Independent core laboratories conducted analyses of and adjudicated angiographic images (SynvaCor, Springfield, Illinois) and duplex ultrasound images (VasCore, Massachusetts General Hospital, Boston, Massachusetts). An independent data safety monitoring board consisting of a biostatistician and physicians not involved in the study reviewed ongoing study data. The study was registered on clinicaltrials.gov under the identifier NCT01366482.

### Patients

Patients from 10 hospitals in Belgium, Germany, Poland, and Switzerland were enrolled. Operators were trained in adequate use of the atherectomy device and had to submit 25 cases for review before they were allowed to participate in the study to guarantee comparable skill sets for all operators. Angiographic eligibility was determined based on visual estimation by the investigator at the time of the procedure. The key general and angiographic inclusion and exclusion criteria are provided in Table 1. Patients with severe calcification in the target lesion, defined as fluoroscopic dense circumferential calcification extending >5 continuous centimeters, were excluded from the randomization but were eligible for the nonrandomized (NR) treatment arm after meeting all other inclusion criteria and no other exclusion criteria.

**Table 1.**
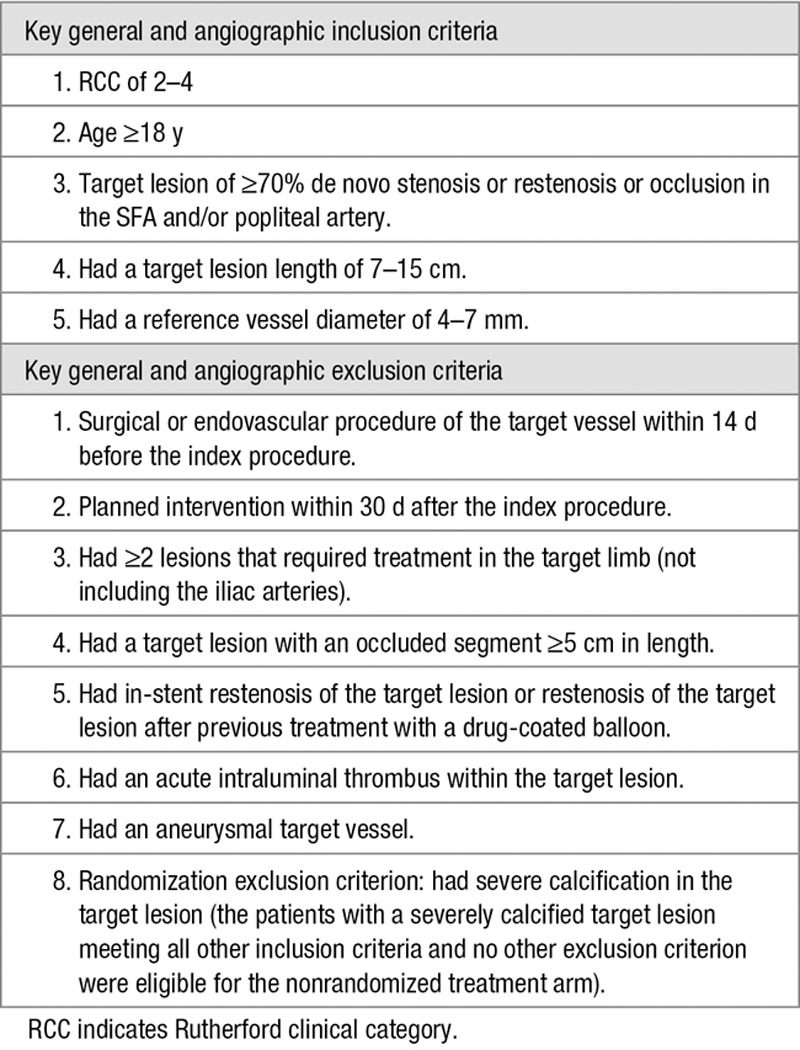
Key Inclusion and Exclusion Criteria

### Randomization

Patients were enrolled by the investigators and randomly assigned to treatment groups after successful passage of the guidewire across the target lesion. Block randomization by center was used to assign patients in a 1:1 fashion to (1) DA+DCB or (2) DCB alone. Crossover to the other treatment arm was not allowed. Patients who were not eligible for randomization because of severe calcification were eligible for enrollment in an NR treatment arm of DA+DCB angioplasty (NR DA+DCB). Although it was not possible to blind investigators, patients, and the angiographic core laboratory to the treatment assignment, the duplex ultrasound core laboratory staff and the clinical events committee were blinded to the treatment assignment.

### Study Devices

DA was performed using the SilverHawk or TurboHawk devices (Medtronic plc, formerly Covidien/ev3, Plymouth, MN). The SilverHawk and TurboHawk devices consist of 2 major components, the SilverHawk or TurboHawk peripheral catheter and the SilverHawk cutter driver. Used together, the system is designed for the treatment of de novo and restenotic atherosclerotic lesions located in native peripheral arteries. All commercially available SilverHawk and TurboHawk catheter models were allowed in the study and the use of distal protection devices was left to the discretion of the operators.

The paclitaxel-coated balloon used in the study was the 0.035″ Cotavance paclitaxel-coated balloon catheter (Bayer HealthCare, formerly MEDRAD, now Medtronic), with the Paccocath coating consisting of a paclitaxel coating concentration of 3 μg/mm^2^ and Ultravist excipient. Paclitaxel promotes the assembly of microtubules from tubulin dimers and stabilizes microtubules by preventing depolymerization. This stability results in the inhibition of the normal dynamic reorganization of the microtubule network that is essential for vital interphase and mitotic cellular functions. It was intended for mechanical/balloon dilatation of stenotic lesions in the iliac and infrainguinal arteries while applying paclitaxel to inhibit restenosis. The Cotavance catheters received Conformité Européene mark approval on August 4, 2011 but are no longer commercially available. The Cotavance balloon was chosen because it was the only drug-coated balloon that had published proof-of-concept data at the time of trial design.^7,8^

The use of >1 study balloon per patient was permitted if required to cover the entire length of a longer lesion. Predilatation with an undersized uncoated angioplasty balloon at low pressure was recommended to allow successful advancement of the DA device or DCB in lesions where a DA device or DCB was unable to cross.

### Procedures

Treatment of only one target lesion per patient, with 3 cm or less between diseased segments requiring treatment, was allowed during the index procedure. Iliac artery lesions could be revascularized before enrollment, but treatment of any other nontarget lesions was not allowed during the index procedure. During the procedure, angiographic imaging, including run-off to the ankle/foot, was required pretreatment, after predilatation or after DA (if performed), after treatment with the DCB, and after adjunctive therapies. In the event of a flow-limiting dissection, perforation, or occlusive complication (eg, recoil), a prolonged angioplasty with an uncoated balloon (5 minutes) was suggested. All efforts were made to reduce the need for bail-out bare metal stent placement. In cases of suboptimal results after prolonged balloon inflation bail-out bare metal stenting was allowed. Adjunctive treatment with cutting balloons or scoring balloons was not allowed. Lesion characteristics were analyzed according to the angiographic core laboratory assessment.

Additional study assessments included RCC, ankle–brachial index (ABI), EuroQOL 5 domains, and walking impairment questionnaire (WIQ) at baseline, 30 days, 6 months, and 1 year postprocedure. Duplex ultrasound evaluations were required at the predischarge or 30-day evaluation and at 6 months and 1 year postprocedure. An angiogram was required at the 1-year follow-up. Required antiplatelet medications included aspirin and clopidogrel preprocedure and postprocedure (clopidogrel for at least 4 weeks and aspirin indefinitely); anticoagulants during the procedure were as indicated by the investigator to maintain appropriate activated clotting times.

### End Points and Definitions

The primary outcome was angiographically defined as the target lesion percent diameter stenosis at 1 year postprocedure, defined as the narrowest point of the target lesion divided by the estimated native vessel diameter at that location as determined by the angiographic core laboratory. Secondary outcomes included technical success (defined as ≤30% residual stenosis following the protocol-defined treatment, before adjunctive treatments, at the target lesion as determined by the angiographic core laboratory) and major adverse event (MAE) rate at 30 days and 1 year, defined as major unplanned amputation of the treated limb, all-cause mortality, or clinically driven TLR. Clinically driven TLR (CD-TLR) was defined as any reintervention or surgical revascularization involving the target lesion in which the patient had ≥70% diameter stenosis and at least 2 of the following: worsening RCC, worsening WIQ score, or an ABI drop >0.15 from baseline and was assessed at 6 months and 1 year. Patency was assessed by duplex ultrasound at 6 months and 1 year with rates calculated using both peak systolic velocity ratio (PSVR) ≤3.5 and PSVR ≤2.4 at the target lesion with no clinically driven reintervention within the target lesion. Measurements at both PSVR values were taken, but PSVR values ≤2.4 were reported here because they are more clinically relevant. Core laboratory assessed angiographic patency at 1 year was determined in all patients. The protocol defined patency as ≤70% stenosis; the more conservative patency of ≤50% stenosis is reported here for consistency with other publications. The 1-year angiographic analysis also included determination of the target lesion minimum lumen diameter and the net luminal gain and difference between baseline and 1-year minimum lumen diameter. Changes in RCC, ABI, EuroQOL 5 domains, and WIQ were also evaluated at 6 months and 1 year in comparison with baseline. The ABI evaluation only included patients with compressible arteries and baseline ABI <0.9.

### Statistical Analysis

All statistical analyses were performed using SAS for Windows (version 9.2; SAS Institute, Inc, Cary, NC). Descriptive statistics have been used to present the data and to summarize the results. Discrete variables are presented using frequency distributions and cross tabulations. Continuous variables are summarized using the number of observations, mean, SD, median, 25th quantile, 75th quantile, minimum, and maximum. Unless otherwise specified, data for all study patients are presented by treatment arm and include all available data for all patients enrolled.

All clinically relevant baseline demographics and procedure characteristics were tabulated for the randomized and NR treatment arms. For categorical variables, differences between the randomized arms were evaluated with Fisher exact test. For continuous variables, the differences were tested using the nonparametric Kruskal–Wallis test.

For longitudinal outcomes measured at multiple follow-up visits, differences between randomized groups over time were evaluated with repeated measures analysis. Logistic regression with generalized estimating equations was performed for the binary duplex patency outcome (PSVR ≤2.4), and linear mixed models were used for the continuous, functional outcomes. A nonparametric approach was performed for EuroQOL 5 domains index, WIQ pain score, and WIQ stair climbing score with linear mixed model analysis based on the rank-transformed outcomes. For the ordinal Rutherford outcome, generalized estimating equations–cumulative logistic regression analysis was performed. It is acknowledged that these analyses are for exploratory purposes only because the study is not designed to have adequate power for such analyses.

For MAE reporting, the outcome analysis is based on patient counts. A patient with >1 event was counted only once toward the event rate based on the total number of patients with MAEs. Event counts are also presented. Kaplan–Meier analysis of the composite MAE was also performed with occurrence of first event used as the basis for event times. Every patient was treated per the randomization, and all analysis was intent-to-treat.

## Results

Between August 2011 and May 2013, 121 patients were enrolled in the DEFINITIVE AR study, with 102 patients randomized to treatment with DA+DCB (n=48) or DCB (n=54). The remaining 19 patients with severely calcified target lesions were treated as NR DA+DCB. TurboHawk devices were used exclusively in the NR severe calcification arm followed by DCB, whereas either SilverHawk (n=18) or TurboHawk (n=37) devices were used in the randomized DA+DCB arm.

### Patient Characteristics

The mean age was 69.6±8.9 years, and 67.8% (82/121) of patients were men (Table 2). All arms of the trial had similar risk factors and comorbidities, including diabetes mellitus and chronic kidney disease. No baseline patient characteristics were statistically significantly different between randomized treatment groups. At study completion, 100% (121/121) of patients completed the predischarge visit, 95.0% (115/121) the 30-day visit, 90.9% (110/121) the 6-month visit, and 88.4% (107/121) the 1-year visit (Figure). The 1-year follow-up completion was 85.4% for the DA+DCB group (3 withdrew, 2 were lost to follow-up, and 2 died), 92.6% for the DCB group (3 withdrew and 1 died), and 84.2% for the NR DA+DCB group (1 withdrew, 1 was lost-to-follow-up, and 1 died).

**Table 2.**
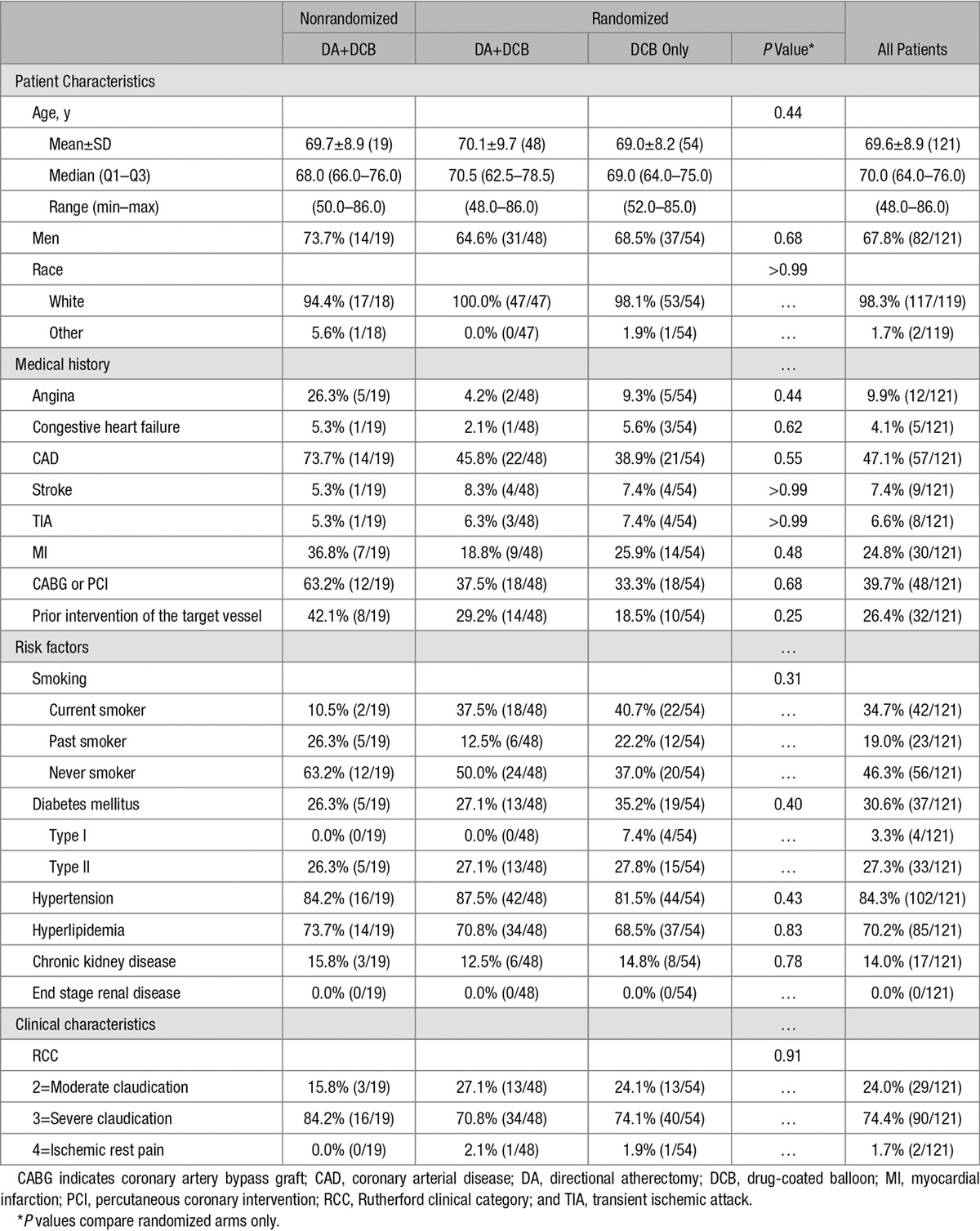
Baseline Demographics, Risk Factors, and Medical History

**Figure.**
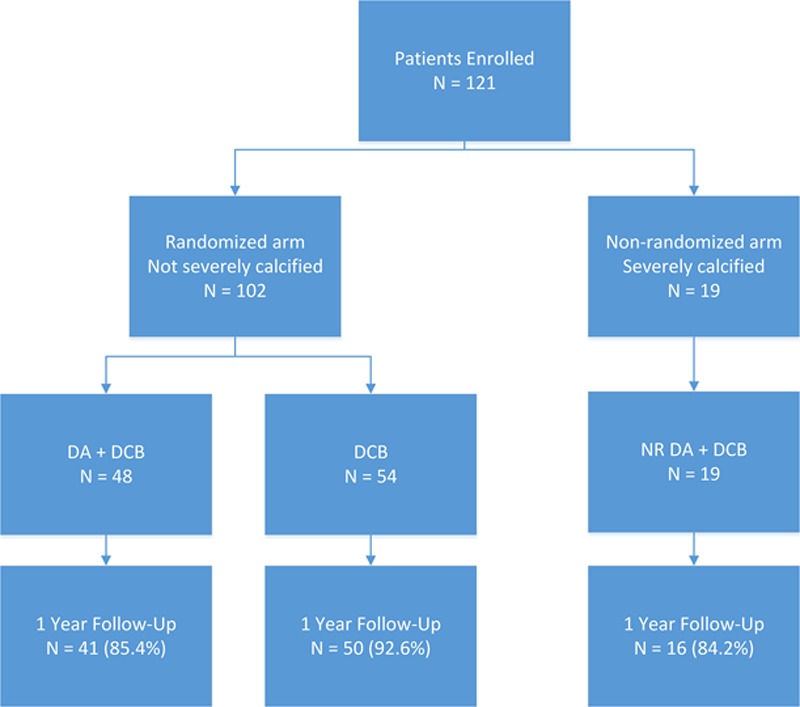
Study profile/compliance. DA indicates directional atherectomy; DCB, drug-coated balloon; and NR, nonrandomized.

### Lesion Characteristics

The mean lesion length as measured by the angiographic core laboratory for all enrolled patients was 106.3±43.9 mm (Table 3). The lesion lengths were longer in the DA+DCB group in comparison with the DCB group (112.3±40.3 mm versus 96.6±40.9 mm; *P*=0.05). The mean lesion length in the NR DA+DCB group was longer than both randomized groups (118.7±56.2 mm). Calcium was present in most lesions treated (70.8% of DA+DCB, 74.1% of DCB, and 100% of NR DA+DCB) as determined by angiography films analyzed by the core laboratory. Severe calcification was found in the NR DA+DCB group (94.7%) and in the randomized arms (25.0% of DA+DCB group versus 18.5% of DCB group). The mean percent diameter stenosis at baseline was 81.9±16.0% for the DA+DCB group, 84.9±14.9% for the DCB group, and 87.9±11.3% for the NR DA+DCB group (Table 3). The majority of lesions were de novo in all arms (89.6% in the DA+DCB group, 92.6% in the DCB group, and 78.9% in the NR DA+DCB group). There was no significant difference between the study arms with regards to lesion calcification, severe calcification, mean percent diameter stenosis, or lesion de novo status.

**Table 3.**
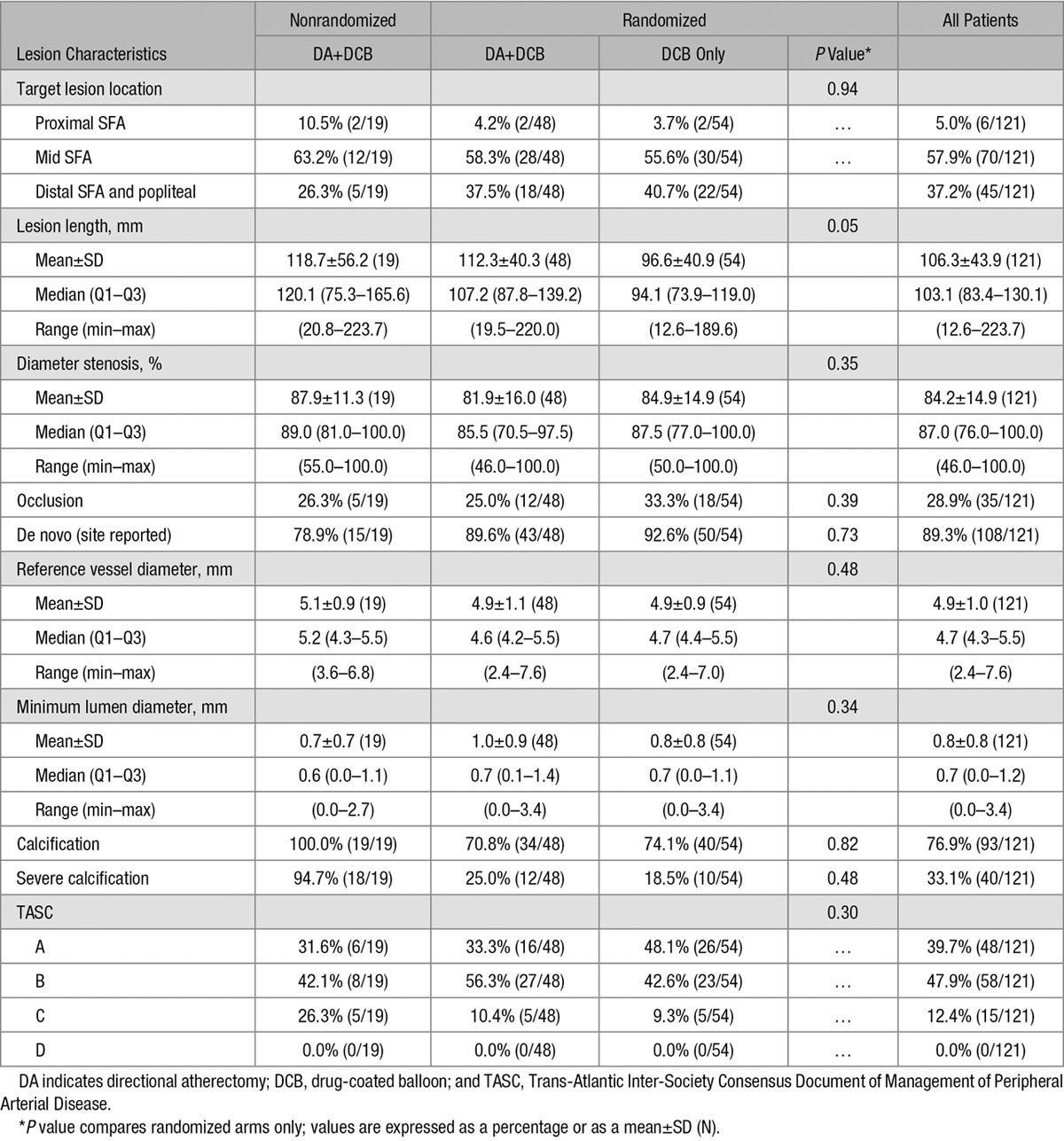
Angiographic Core Laboratory Reported Lesion Characteristics

### Procedural Outcomes

Technical success (≤30% residual stenosis following protocol-assigned treatment, before adjunctive treatments) at the target lesion was 89.6% for the DA+DCB group versus 64.2% for the DCB group (*P*=0.004). Technical success in the NR DA+DCB group was 84.2%. Use of the SpiderFX filter for distal embolic protection was reported in patients treated with atherectomy; 85.4% of DA+DCB patients and 100% of NR DA+DCB patients had a SpiderFX filter in place during the procedure.

Of the randomized patients, 16.7% of the DA+DCB patients received predilation before use of the DA device in comparison with 74.1% in the DCB-only arm. Predilatation occurred in 31.6% of patients enrolled in the NR DA+DCB arm. Adjunctive therapies after DA included bail-out stenting and postdilation. In the randomized DA+DCB arm, 6.3% of patients received adjunctive therapy, compared with 37.0% in the DCB alone arm (*P*<0.0001). In the NR DA+DCB group, 5.3% of patients received adjunctive therapy. Bail-out bare metal stenting was performed for 2 DCB patients (3.7%) and 1 NR DA+DCB patient (5.3%), and postdilatation PTA was performed for 3 DA+DCB patients (6.3%) and 18 DCB patients (33.3%; *P*=0.001).

The most common procedural complications (Table 4) were grade C/D dissections, which occurred more frequently in the DCB arm (n=10; 18.5%) than in the DA+DCB group (n=1; 2.1%; *P*=0.01). No relevant dissections occurred in the NR DA+DCB group. In the DA+DCB cohort, there were 2 clinically significant distal embolization events requiring endovascular interventions and 1 distal embolization event that was not clinically significant. Additionally, 2 perforations (4.2%) occurred in the DA+DCB group with none in the DCB group (*P*=0.22). Both perforations were successfully treated with prolonged PTA; however, per protocol, treatment with DCB was not allowed after the perforations.

**Table 4.**
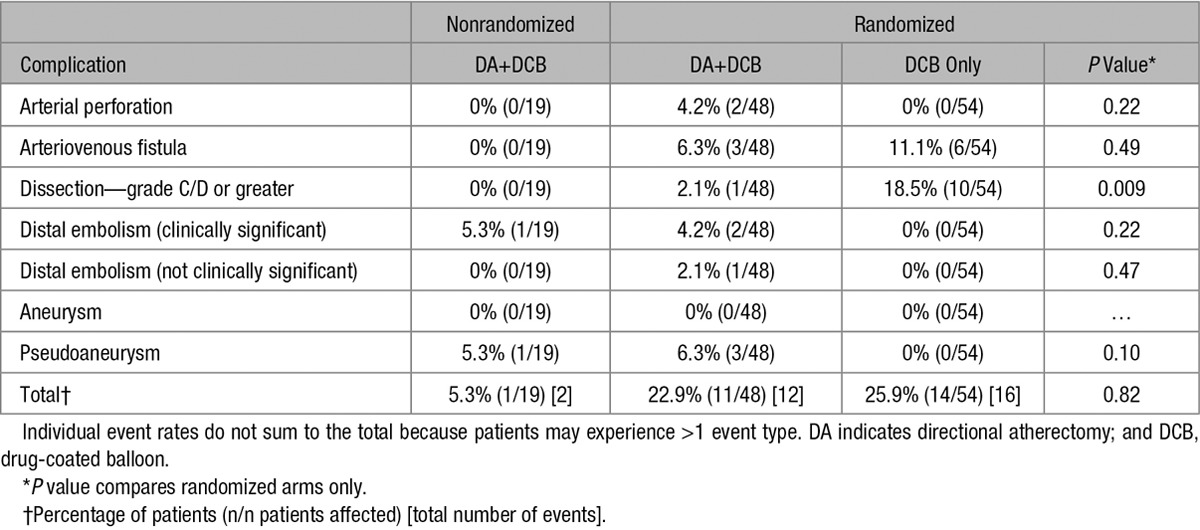
Procedural Complications

### Angiographic and Duplex Sonographic 1-Year Outcomes

The 1-year primary end point of angiographic target lesion percent diameter stenosis was 33.6±17.7% (n=33) for the DA+DCB group versus 36.4±17.6% (n=39) for the DCB group (*P*=0.48) and 55.0±30.5% (n=14) for the NR DA +DCB group (Table 5). For lesions ≥10 cm, the 1-year mean percent diameter stenosis was 31.1±11.9% for the DA+DCB group (n=20) and 36.6±13.7% for the DCB group (n=16). No significant differences were found in the minimum lumen diameter and net lumen gain at 1 year (Table 5; *P*=0.48 and 0.62).

**Table 5.**
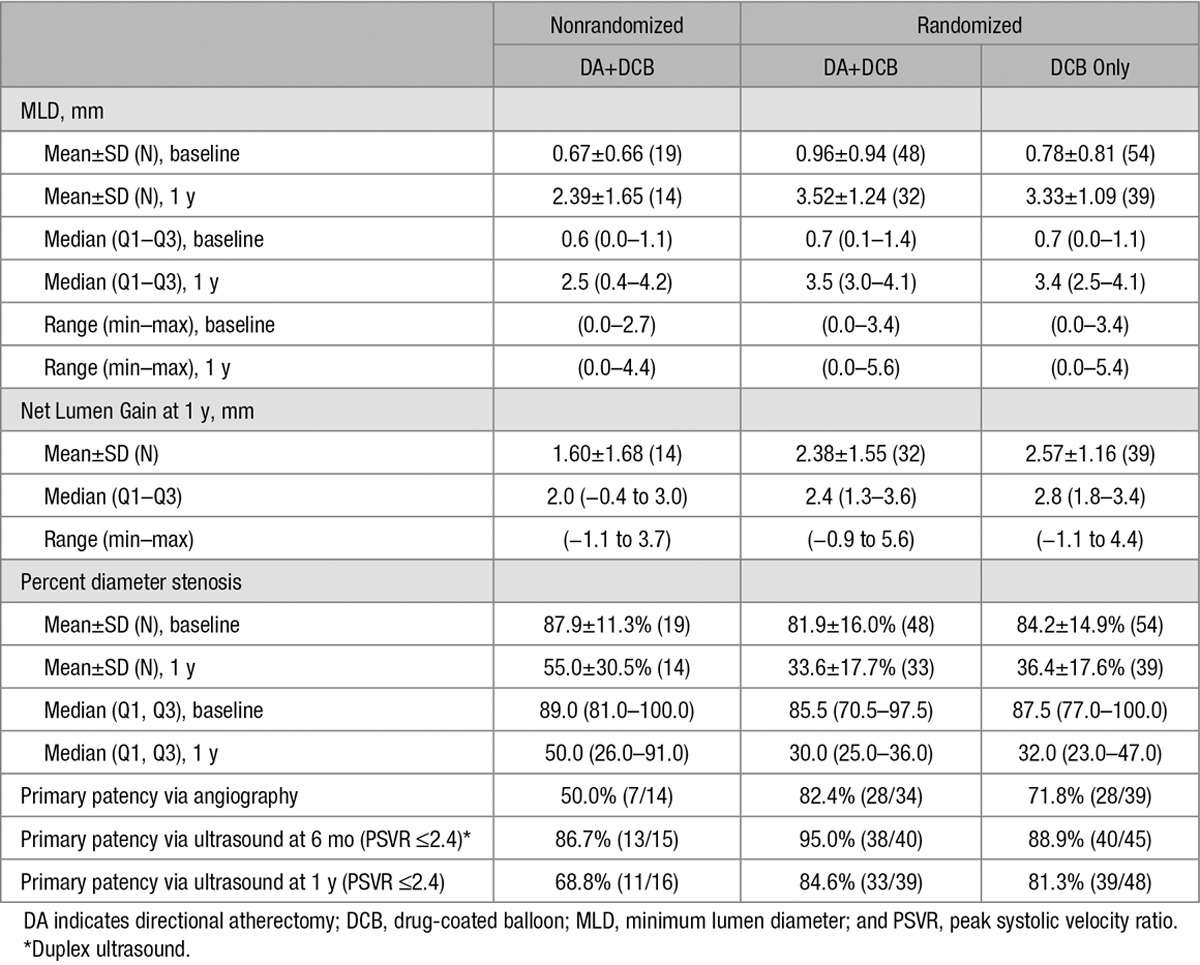
Vessel Characteristics and Results

The 1-year angiographic primary patency was 82.4% (28/34) for the DA+DCB group versus 71.8% (28/39) for the DCB group (*P*=0.41) and 50.0% (7/14) for the patients in the NR DA+DCB cohort. Patency was also assessed via duplex ultrasonography (DUS). The DUS patency rate at 6 months and 1 year, respectively, was 95.0% (38/40) and 84.6% (33/39) for the DA+DCB group, 88.9% (40/45) and 81.3% (39/48) for the DCB group (*P*=0.44 at 6 months and *P*=0.78 at 1 year), and 86.7% (13/15) and 68.8% (11/16) for the NR DA+DCB group. Using all available DUS patency data at 6 and 12 months, no significant differences over time were observed between randomized groups (generalized estimating equations logistic regression *P*=0.42). The patency rate using PSVR ≤3.5 at 6 months and 1 year, respectively, was 95.0% (38/40) and 87.2% (34/39) for the DA+DCB group, 93.3% (42/45) and 89.6% (43/48) for the DCB-only group (*P*>0.99 at 6 months and *P*=0.75 at 1 year), and 86.7% (13/15) and 75.0% (12/16) for the NR DA+DCB group.

An ad hoc angiographic analysis of patency in lesions >10 cm demonstrated a trend toward a potential advantage to DA+DCB treatment of 95.0% for DA+DCB (n=20) versus 68.8% for DCB (n=16). However, this trend was less evident in the 1-year duplex ultrasound patency analysis: (PSVR≤2.4) of 95.7% for DA+DCB (n=23) versus 85.0% for DCB (n=20). A trend toward achieving better 12-month patency was seen related to achieving ≤30% residual stenosis post-DA before DCB treatment. For lesions in the DA+DCB group that achieved ≤30% residual stenosis after DA, angiographic 1-year patency was 88.2% (n=17) and DUS 1-year patency (PSVR≤2.4) was 84.2% (n=19) in comparison with 68.8% (n=16) and 77.8% (n=18) for lesions with >30% residual stenosis after DA.

The data also suggest a potential advantage of DA+DCB for severely calcified lesions, but the number of lesions was too small to show statistical significance. All lesions were reclassified as either severely calcified or not severely calcified by the core laboratory, regardless of their inclusion in either the DA+DCB, DCB only, or NR DA+ DCB arm. Based on this classification, 1-year patency by angiography of severely calcified lesions treated with both DA and DCB was 58.3% (14/24), and patency of severely calcified lesions treated with DCB alone was 42.9% (3/7). For this same grouping of patients, DUS patency at 1 year was 70.4% (19/27) for patients treated by DA and DCB versus 62.5% (5/8) for patients treated by DCB alone.

### Major Adverse Events

MAEs were defined as major unplanned amputations of the treated limb, all-cause mortality, or CD-TLR. A total of 18 MAEs were reported and adjudicated by the clinical events committee, including 4 deaths and 14 CD-TLRs. No major amputation was observed. The 30-day freedom from MAE rate determined by Kaplan–Meier was 97.9% for DA+DCB, 98.1% for DCB, and 100.0% for NR DA+DCB. The 30-day MAEs included a single CD-TLR in each of the randomized study arms. The 1-year freedom from MAE rate determined by Kaplan–Meier was 89.3% for DA+DCB versus 90.0% for DCB (*P*=0.86) and 94.4% for NR DA+DCB. The 1-year MAE events included 2 deaths and 3 CD-TLRs after DA+DCB, 1 death and 5 CD-TLRs in the DCB arm, and 1 death in the NR DA+DCB group. Differences in the MAE rates at both 30 days and 1 year were not statistically significant. No amputations of the treated limb occurred. None of the 4 deaths were attributed to the study devices or procedure (heart failure/stroke, acute coronary syndrome, respiratory failure, and neoplastic disorder).

At 6 months, 4.7% (2/43) of the DA+DCB group had a CD-TLR compared with 3.9% (2/51) of the DCB group. At 1 year, 7.3% (3/41) of the DA+DCB group had a TLR compared with 8.0% (4/50) of DCB group. No CD-TLR occurred in the NR DA+DCB group. The Kaplan–Meier-estimated freedom from CD-TLR rates were 95.7% versus 96.3% at 6 months and 93.2% versus 91.9% at 1 year for the DA+DCB and DCB groups, respectively, and 100% at 6 months and 1 year for the NR DA+DCB group.

### Functional Outcomes

No significant differences in functional outcomes were observed between the randomized groups (Table 6). The majority of patients had an improvement of at least 1 RCC at 6 months (97.7% DA+DCB, 90.2% DCB, and 87.5% NR DA+DCB) and at 1 year (85.4% DA+DCB, 93.9% DCB, and 81.3% NR DA+DCB) in comparison with baseline. Only one patient (DCB group) had a worsening of RCC at 6 months. Similarly, improvement in ABI for patients with compressible arteries and baseline ABI<0.9 were observed for 85.2% DA+DCB, 85.7% DCB, and 88.9% NR DA+DCB of patients at 6 months and 89.3% DA+DCB, 88.6% DCB, and 88.9% NR DA+DCB of patients at 1 year. Improvements in WIQ and EuroQOL 5 domain scores were also seen in all groups at 6 months and 1 year.

**Table 6.**
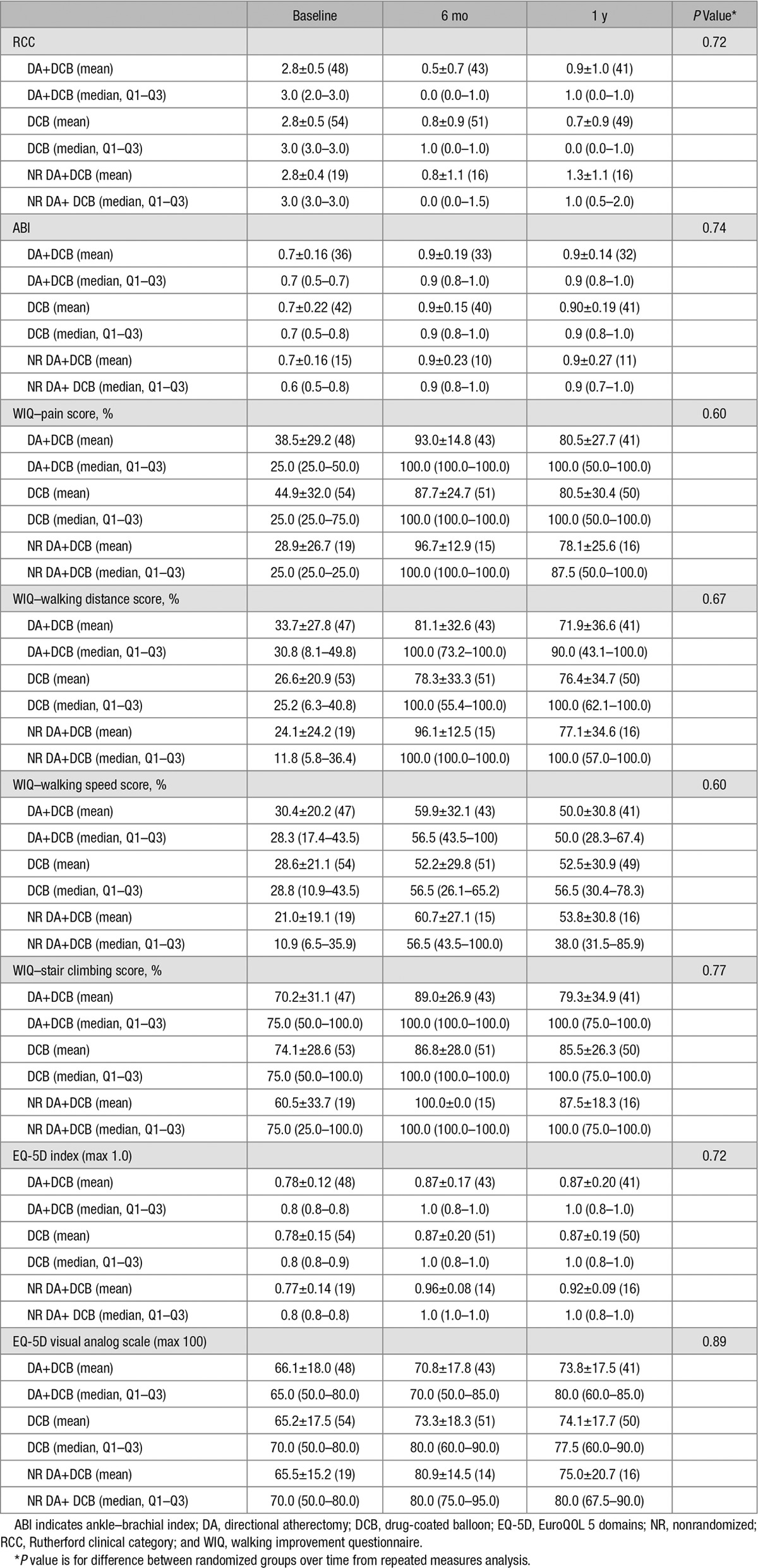
Functional Outcomes

## Discussion

DEFINITIVE AR was the first prospective, multicenter study designed to estimate the effect of treating femoropopliteal arteries with DA before a DCB. Because this was a pilot study designed to facilitate development of additional studies, differences in outcomes show only nonsignificant trends because of lack of cohort size to yield statistical power. Major findings of DEFINITIVE AR include the safety of the combination of DA with DCB ≤1-year follow-up and potential patency benefits for both longer and severely calcified lesions. This is especially relevant to the field because using PTA alone to treat both longer and calcified lesions remains a challenge. DA is well suited to remove plaque and to potentially reduce vessel recoil, reduce the rate of severe dissections, and facilitate a more homogenous drug application and diffusion into the vessel wall layers.^15,21^ Combining a device that reduces plaque volume with one that reduces neointimal hyperplasia (DA+DCB) may be an even more effective treatment of lower extremity PAD.

DA that resulted in <30% stenosis was associated in a post hoc analysis with a higher 1-year patency rate whether patency was measured by angiography or duplex. Lesions with ≤30% residual stenosis after DA resulting in lumen gain achieved angiographic patency of 88.2% and duplex patency of 84.2%, whereas lesions with >30% residual stenosis achieved angiographic patency of 68.8% and duplex patency of 77.8%. In the randomized DA+DCB arm, no stents were required, and only one lesion required stenting in the registry arm, confirming the findings of the DEFINITIVE LE (Determination of Effectiveness of the SilverHawk® Peripheral Plaque Excision System (SilverHawk Device) for the Treatment of Infrainguinal Vessels / Lower Extremities) and the DEFINITIVE calcium studies with stent rates after DA of 3.0% and 4.1%, respectively.^15,21^ All efforts were made to reduce the need for bail-out bare metal stent placement. Because the use of reentry devices was an exclusion criterion, most of the occlusions were crossed intraluminally, potentially resulting in a reduced likelihood of multiple dissection planes, which usually result in bail-out stent placement, potentially explaining the low overall bail-out stent rate in the present study.

The 1-year DUS patency rates of 84.6% for the randomized DA+DCB arm in DEFINITIVE AR confirms the outcome of a small prospective, single-center study conducted by Cioppa et al^17^ of patients with severely calcified femoropopliteal lesions (defined as fluoroscopic calcification on both sides of vessel wall >1 cm in length). Patients were treated with DA until a <30% residual stenosis was achieved, as confirmed by intravascular ultrasound and angiography, after by DCB (IN.PACT Admiral, Medtronic, Inc). In their study, the bail-out stent rate was 6.5%, the 1-year duplex-derived primary patency rate was 90%, and the freedom from MAEs was 87%. Both this study and the current study highlight the potential value of DA+DCB in calcified lesions.

Stavroulakis et al^18^ measured patency, procedural success, morbidity, and mortality after treatment of popliteal lesions with a combined therapy of DA and DCB. In their single-arm study of 21 patients, 1-year primary patency was 95% as estimated by Kaplan–Meier.

The outcome of the DCB-only arm of the present study confirms the performance of the DCB arms of the historical THUNDER trial (Local Taxan With Short Time Contact for Reduction of Restenosis in Distal Arteries) and FEMPAC trial (Femoral Paclitaxel) where similar DCBs were used.^7,8^ Because of the excellent performance of DCB alone in TASC II A and B femoropopliteal lesions—provided they are not severely calcified—the DA+DCB strategy should be reserved to more complex lesions.

Currently, there is no published economic analysis of the use of DA+DCB. Panaich et al^22^ reported that although atherectomy utilization was predictive of lower in-hospital mortality, lower amputation rates, and lower complication rates, it was also associated with higher cost of procedures in 2012 (although authors noted the population of patients treated by atherectomy tended to have more diffuse disease and comorbidities). Pietzsch et al^23^ concluded that DCB technology is associated with lower revascularization rates and cost savings for both the United States and Germany. The recently published DCB budget impact model goes so far as to assert that “DCBs offer the highest clinical and economic value.”^24^ Considering worldwide limited financial resources for healthcare systems, cost-effectiveness analyses should be implemented into future larger-scale randomized controlled trials evaluating the true benefit of combining DA+DCB. An ideal follow-up study would include an increased number of patients, longer-term follow-up, and focus on calcified lesions.

### Limitations

The study was not sufficiently powered as a pivotal study to draw final conclusions about the impact of DA for lesion preparation before DCB angioplasty in femoropopliteal interventions. Additionally, there was a significant difference in balloon pre- and postdilatation between trial arms. Finally, the mean lesion length in the DA+DCB group was longer than the DCB group, potentially preventing detection of a significant difference between the groups. A follow-up study should include an increased number of patients, a focus on calcified lesions, and patients should be followed up for a longer period of time. Moreover, the outcomes cannot be extrapolated to the impact of other atherectomy or DCB technologies, or in other lesion locations.

### Conclusions

Although DCB has been shown to increase primary patency rates in TASC II A and B femoropopliteal lesions, challenging subsets remain: severely calcified lesions do not experience the same patency.^12^ For the treatment of femoropopliteal lesions, vessel preparation with DA before DCB angioplasty seems to be safe in mid-term follow-up and might have benefits in more challenging lesion subsets that are at higher risk for acute and chronic technical treatment failure of PTA, including DCB angioplasty, such as severely calcified lesions. In this study, treatment with DA+DCB resulted in both increased technical success and fewer flow-limiting dissections compared with treatment with DCB alone. A sufficiently powered randomized controlled trial is needed to further characterize the potential benefits of DA+DCB.

## Acknowledgments

We would like to recognize and thank the patients involved with this clinical study for their participation. We acknowledge the following individuals from Medtronic for their contributions: Mei Jiang for statistical analysis and Azah Tabah, PhD, and Bridget Wall, PhD, for their medical writing support.

## Sources of Funding

The study was an investigator-initiated study with industry funding from Covidien (now Medtronic PLC, Santa Rosa, CA). Study cohort size was limited by funding.

## Disclosures

Dr Zeller reports that he is a member of the advisory board of Medtronic (Covidien), Spectranetics, Veryan, Boston Scientific, W.L. Gore, and TriReme. Dr Rocha-Singh reports that he is a consultant for Medtronic (Covidien), Alucent, and Zimmer-Biomet and a member of the Board of Vascular Interventional Advances Physicians—a 501c3 not-for-profit education and research organization. Dr Jaff reports that he is a noncompensated advisor for Medtronic (Covidien) and a compensated member of the Board of Vascular Interventional Advances Physicians. Dr Blessing reports that he receives speaker’s honoraria for Medtronic. Dr Scheinert reports that he is on the advisory board and acts for a consultant for Abbott, Biotronik, Boston Scientific, Cook Medical, Cordis, CR Bard, Gardia Medical, Medtronic (Covidien), TriReme Medical, Trivascular, and Upstream Peripheral Technologies. Dr Langhoff reports that he receives speaker’s honoraria for Medtronic and that he is consultant for Biotronik, B. Braun Melsungen, Boston Scientific, dp-medsystems, Contego Medical, Terumo, and Abbott Vascular. Dr Tepe receives Medtronic study support and is a member of the advisory board. The other authors report no conflicts.
